# Role of Defect Engineering and Surface Functionalization in the Design of Carbon Nanotube-Based Nitrogen Oxide Sensors

**DOI:** 10.3390/ijms222312968

**Published:** 2021-11-30

**Authors:** Manuel A. Valdés-Madrigal, Fernando Montejo-Alvaro, Amelia S. Cernas-Ruiz, Hugo Rojas-Chávez, Ramon Román-Doval, Heriberto Cruz-Martinez, Dora I. Medina

**Affiliations:** 1 Instituto Tecnológico Superior de Ciudad Hidalgo, Tecnológico Nacional de México, Av. Ing. Carlos Rojas Gutiérrez 2120, Fracc. Valle de la Herradura, Ciudad Hidalgo 61100, Mexico; mavm8405@hotmail.com; 2 Instituto Tecnológico Del Valle de Etla, Tecnológico Nacional de México, Abasolo S/N, Barrio Del Agua Buena, Santiago Suchilquitongo, Oaxaca 68230, Mexico; moaf1217@gmail.com (F.M.-A.); rrdoval.11@gmail.com (R.R.-D.); 3 Instituto Tecnológico del Istmo, Tecnológico Nacional de México, Panamericana 821, 2da., Juchitán de Zaragoza, Oaxaca 70000, Mexico; amelia.cr@istmo.tecnm.mx; 4 Instituto Tecnológico de Tláhuac II, Tecnológico Nacional de México, Camino Real 625, Tláhuac, Ciudad de México 13508, Mexico; rojas_hugo@ittlahuac2.edu.mx; 5 School of Engineering and Sciences, Tecnologico de Monterrey, Atizapan de Zaragoza 52926, Mexico

**Keywords:** experimental studies, DFT calculations, adsorption, selectivity, sensibility

## Abstract

Nitrogen oxides (NO_x_) are among the main atmospheric pollutants; therefore, it is important to monitor and detect their presence in the atmosphere. To this end, low-dimensional carbon structures have been widely used as NO_x_ sensors for their outstanding properties. In particular, carbon nanotubes (CNTs) have been widely used as toxic-gas sensors owing to their high specific surface area and excellent mechanical properties. Although pristine CNTs have shown promising performance for NO_x_ detection, several strategies have been developed such as surface functionalization and defect engineering to improve the NO_x_ sensing of pristine CNT-based sensors. Through these strategies, the sensing properties of modified CNTs toward NO_x_ gases have been substantially improved. Therefore, in this review, we have analyzed the defect engineering and surface functionalization strategies used in the last decade to modify the sensitivity and the selectivity of CNTs to NO_x_. First, the different types of surface functionalization and defect engineering were reviewed. Thereafter, we analyzed experimental, theoretical, and coupled experimental–theoretical studies on CNTs modified through surface functionalization and defect engineering to improve the sensitivity and selectivity to NO_x_. Finally, we presented the conclusions and the future directions of modified CNTs as NO_x_ sensors.

## 1. Introduction

Novel technologies have undoubtedly allowed human civilization to reach a rapid development stage, which is mainly associated with the rapid industrialization of many countries. Approximately 75% of the global energy consumption used to achieve this was nonrenewable; that is, the energy requirements were supplied mainly from fossil fuels [1]. It is no overstatement to say that toxic emission constituents depend upon the incomplete combustion of hydrocarbons, which results in several by-products, such as O_x_, CO_x_, HO_x_, SO_x_, PO_x_, RO_x_, MO_x_, and NO_x_ [2]. For this reason, although fossil fuels are limited in quantity, they have harmed the environment irreparably, despite governments implementing tax policies to discourage their use [3,4,5]. Accordingly, NO_x_, among many other pollutants, is a component of our atmosphere that has considerably decreased the air quality around us. In this context, air pollution has direct and indirect effects on the human health, ecosystems, and climate, with consequent economic and social costs. For example, in the last two decades, health expenditures have increased due to air pollution [3].

Lamentably, air pollution constitutes a major problem in urban areas. In this sense, nitrogen oxides (NO_x_) are among the primary air pollutants. Anthropogenic NO_x_ is formed during combustion processes at high temperatures during the operation of motor vehicles and various industrial activities [3,6]. Automotive exhaust is the main source for NO_x_ in urban areas. Several statistical epidemiological studies have associated air pollution with human health and mortality. For instance, air containing large amounts of NO_x_ can cause respiratory problems in the elderly, children, and patients with asthma [3]. Furthermore, NO_x_ has been recognized as an important factor in the deterioration of materials.

Air quality still affects the health of the population and perpetuates environmental degradation, e.g., the disruption of ecological balance and climate change. According to emissions projections from the World Health Organization, a massive increase in air pollution will lead to increased premature mortality caused by environmental degradation by 2050 [3]. Therefore, it is important to monitor air pollution by NO_x_, in addition to other pollutants. Undoubtedly, accurate measurement of NO_x_ exposure in any given area, which is a demanding task, is required. From a theoretical and experimental perspective, this demonstrates the necessity of developing new sensors for NO_x_ detection. Therefore, the detection of toxic gases has become an important field of research.

It is not surprising that many nanomaterials have been proposed to detect such pollutants [7,8]. Even though toxic-gas sensors are conventionally designed and manufactured using semiconducting oxides (e.g., ZnO [9], SnO_2_ [10], and Fe_2_O_3_ [11]), their use has been limited owing to poor sensibility and selectivity, as well as high operating temperatures [9,10,11]. In addition, it is worth highlighting that toxic-gas sensors, to be used in practice, should fulfil many requirements in terms of purposes and conditions of sensor operation. These are all connected with the aim to save energy, which is of key importance to have a remarkable increment in the toxic-gas sensors’ life [12]. Along with this, the decrease in the power consumption—for gas detection technologies—should allow their fast integration into a wide range of common electronic devices associated to further improvements of modern life services, but it is still a challenging task [12]. To overcome these limitations, carbon nanostructures are currently the most promising materials to achieve such purposes. It is well known that carbon can form several different synthetic allotropes (e.g., fullerene, graphene, and nanotubes) [13], but it also exists as natural structures (e.g., diamond and graphite) that can be interconverted under specific conditions [14,15]. Among these materials, carbon nanotubes (CNTs) have attracted great attention in the design of NO_x_ sensors. In fact, the properties of CNTs have become active fields in modern research on new materials for toxic-gas sensors.

CNTs were first reported in the seminal work of Iijima in 1991 [16]. These nanomaterials are attractive for their interesting properties and possible applications as sensors for toxic gases. For instance, these exhibit fascinating properties, such as superior electrical conductivity [17,18], large surface area [19], excellent mechanical flexibility [20], high thermal/chemical stability [21,22], and high electron mobility [23]. Although pristine CNTs have shown promising performance for NOx detection [24,25,26], several strategies have been developed such as surface functionalization and defect engineering to improve the NOx sensing of pristine CNT-based sensors [27,28,29,30,31,32,33,34,35,36,37]. Through these strategies, the sensing properties of modified CNTs toward NO_x_ gases have been substantially improved [38,39,40]. Therefore, the modification of CNTs via surface functionalization and defect engineering is a relevant research field at both the theoretical and experimental levels for designing novel CNT-based NO_x_ sensors. Since CNTs are an interesting subject to be studied, a recent progress in gas sensors based on modified CNTs to detect NO_x_ has been revised in the literature [41,42], but those review articles are mainly focused on experimental findings. To date, there has not been a review article that analyzes the current approaches employing theoretical calculations and combining theoretical–experimental investigations. Therefore, the goal of this review is focused on recent advances (in the last decade) about modified CNTs as a promising material for sensing NO_x_ from both a theoretical and an experimental viewpoint, which allows a progress in the state-of-the-art. First, the types of surface functionalization and defect engineering are explained. After, the different modifications made to the CNTs from the experimental, theoretical, and combined theoretical–experimental studies are reviewed. Finally, we present the conclusions and the current challenges.

## 2. Surface Functionalization and Defect Engineering on CNTs

**Figure 1:**
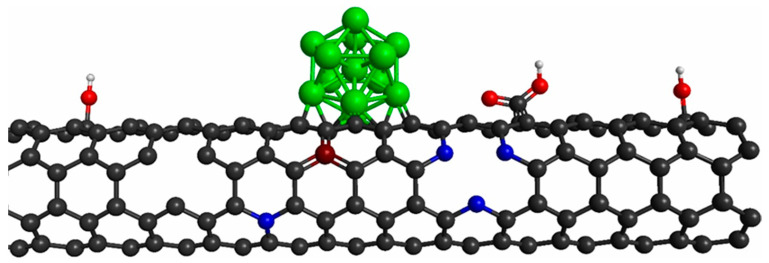
Surface functionalization and defect engineering in CNTs.

The CNTs have been widely studied and used due to their excellent mechanical, thermal, electromechanical, and chemical properties, as well as their high specific surface area [43,44]. They have been used in different fields such as catalysis, sensors, water treatment, electronics, and crop protection [45,46,47,48,49,50]. CNTs are cylindrical molecules that consist of rolled-up sheets of carbon hexagons that can be single (SWCNTs) or multiwall (MWCNTs); normally, the diameter varies from 0.8 to 2 nm and 5 to 20 nm, respectively, and their length reaches a few microns [51,52], which is generally synthesized by chemical vapor deposition (CVD), laser ablation, or electric arc [53,54]. CNTs are composed of strong sp^2^ bonds that provide excellent strength [55], although these strong sp^2^ bonds do not permit good chemical reactivity. Therefore, their application in some fields is limited (e.g., sensor and catalysis fields) [56], and several strategies have been developed to improve their chemical reactivity, such as surface functionalization and defect engineering (Figure 1). These strategies have substantially improved the reactivity of nanotubes to various gases [57,58]. Consequently, CNTs have become promising candidates for applications in the sensor field.

**Figure 2:**
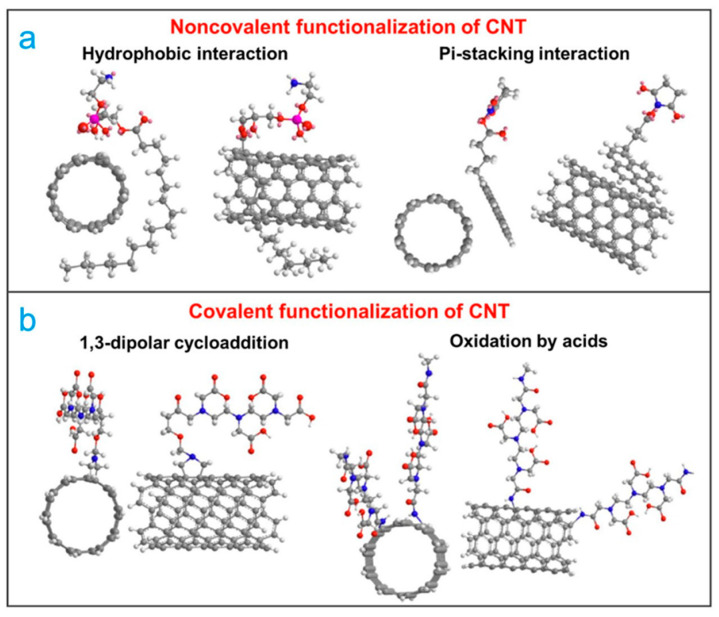
Functionalization modes of CNTs surface. (**a**) noncovalent functionalization and (**b**) covalent functionalization on the CNTs surface [61].

The surface functionalization of CNTs is classified as noncovalent or covalent, where noncovalent functionalization is based on supramolecular complexation via wrapping and adsorptive forces (e.g., π-stacking interactions and van der Waals forces). This type of functionalization does not damage the structure of the sidewall of CNTs. Noncovalent functionalization is commonly used by surfactants and polymers due to the interactions of the hydrophobic part of the adsorbed molecules with nanotubes sidewalls through van der Waals, π–π, CH–π, and other interactions, and aqueous solubility is provided by the hydrophilic part of the molecule [59,60]. This can provide an increase in the solubility and the hydrophilicity of CNTs and help reduce the tendency of CNTs to aggregate. Nevertheless, as a result of the surface functionalization of CNTs by surfactants, their interfacial adhesion is weak [59]. In covalent functionalization, the organic molecules, polymers, or metal nanoparticles are covalently bonded on the surface of CNTs, as shown in Figure 2 [61]. Covalent functionalization of the surface of CNTs can be achieved by adding covalently linked oxygen-containing groups, such as hydroxyl (OH), carbonyl (C = O), and carboxyl groups (COOH) [62]. These groups can be added at the ends, defects, and the sidewall. This chemical modification is achieved by chemical treatment with oxidizing agents such as nitric acid (HNO_3_), sulfuric acid (H_2_SO_4_), hydrochloric acid (HCl), and potassium permanganate (KMNO_4_) [63].

**Figure 3:**
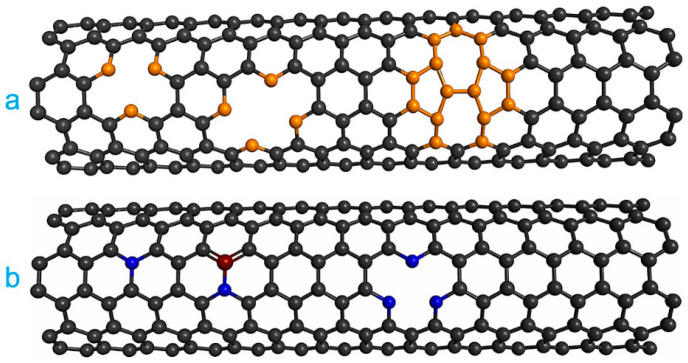
Defect engineering in CNTs. Types of (**a**) vacancies and (**b**) doping.

More recently, defect engineering has become an important method to modify the properties of CNTs [64,65]. Different types of defects have been explored to modify the reactivity of CNTs, including vacancies, substitutional defects (i.e., doping), combined vacancies and substitutional defects, and edge defects. Figure 3a shows the types of vacancies most commonly used to modify the properties of CNTs. Another type of defect widely used is the substitutional defect, which is also known as doping. Doping with a single type of atom or combining two types of atoms in doping have been employed, as shown in Figure 3b, and another strategy is the combination of vacancy and doping. A well-known case of this type of structure is pyridine-type nitrogen doping. It has been reported that defect engineering substantially modifies the electronic and structural properties of pristine CNTs, which causes a substantial improvement in the reactivity of CNT [66,67,68,69].

## 3. Experimental Studies

CNTs have been widely used for NO_x_ sensing [70,71]. In this context, the first studies showed the good performance of pristine CNTs for NO_x_ detection. For instance, Kong et al. demonstrated that the CNTs can be used as chemical gas sensor [25]. In their study, CNTs thin films were deposited onto SiO_2_/Si substrates via chemical vapor deposition technique. The measurements were performed under argon or under an air atmosphere at room temperature (RT); the gas sensors showed fast response and high sensitivity when they were exposed to NO_2_. In another study, Li et al. fabricated a NO_2_ sensor using SWCNTs on gold electrodes [72]. The response of the sensor was up to 0.044 ppm with a recovery time of 10 h. Afterwards, the sensing properties of pristine CNTs on Si_3_N_4_/Si were reported by Valentini et al. [73]. CNTs were synthetized using plasma-enhanced CVD process. The CNTs/Si_3_N_4_ sensors were tested at different temperatures and exhibited a higher sensitivity to NO_2_ at RT. Piloto et al. sensed NO_2_ gas using pristine CNTs films with different thicknesses. They reported a detection limit of 1 ppm and a high sensitivity using a thickness of ≈5 nm, which was tested at RT. This response is attributed to the high density of CNTs [74]. Although these studies have demonstrated the potential of pristine CNTs for NO_x_ detection, several approaches have been used to improve the pristine CNTs sensing properties (e.g., high sensitivity and low operating temperature, fast response, shorter recovery time, high selectivity, easily scalable for mass production and low cost) toward NO_x_ gas such as surface functionalization and defect engineering. Through these approaches, the modified CNTs sensing properties toward NO_x_ gases have been substantially improved. Accordingly, to date, many experimental investigations have been performed using different synthesis methods to improve the NO_x_ detection by CNTs modified through defect engineering and surface functionalization. Therefore, in this section, in last decade, experimental studies on CNTs modified through surface functionalization and defect engineering to improve the sensitivity and selectivity toward NO_x_ are analyzed. These studies are mainly focused on chemiresistive gas sensors.

### 3.1. Functionalized CNTs

**Figure 4:**
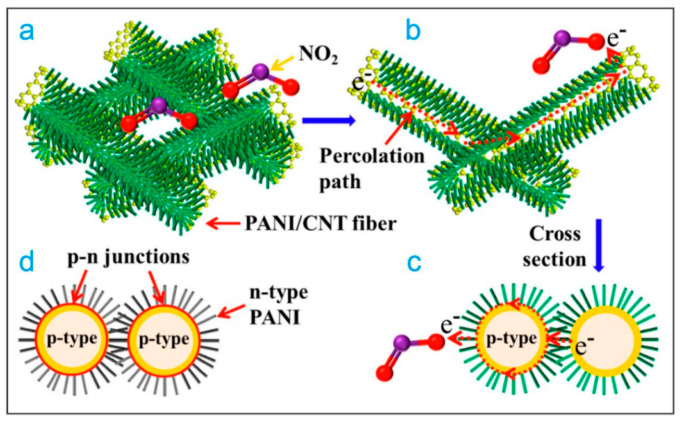
Possible sensing mechanism: (**a**) sketch diagram of conductive network of hierarchical p-PANI/CNT fibers, (**b**) percolation path through conjugate interfaces of PANI and MWCNTs, (**c**) cross-section of PANI/CNT fibers, and (**d**) p-n heterojunction structure of hierarchical n-PANI/CNT fibers [82].

A strategy to improve the sensing of NO_2_ gases is the use of CNTs functionalized by molecules anchored on their surface. Polyaniline (PANI) is a polymer widely used for the functionalization of CNTs owing to its extraordinary electrical properties, chemical stability, the ease of property modification via inorganic acids, low cost, and the ease of synthesis; when interacting with CNTs, it increases the transportation rate of charge carriers [75,76,77,78]. As a result of these properties, PANI is widely used to modify the surface of CNTs. Yun et al. functionalized CNTs using PANI polymerization for NO gas sensing. The samples were tested using TiO_2_ as a catalyst in a vacuum chamber at a pressure of 1 × 10^−6^ mbar, where the sensors were exposed to NO gas at 25 ppm [79]. In another investigation, the PANI and poly(3,4-ethylenedioxythiophene)–polystyrene sulfonic acid (PEDOT:PSS) were used to modify the reactivity of CNTs for NO_2_ gas detection; multiwalled CNTs were synthetized on silicon substrates by the CVD method. PANI and PEDOT:PSS were dissolved using different organic solvents to modify their properties and then spin-coated onto CNTs grown on silicon substrates; a high sensitivity (29.8%) to NO_2_ at 100 ppm was observed for the PANI–MWCNT composite using dimethyl sulfoxide at RT [80]. Using the same approach, PANI modified with sulfonic acid has been used to functionalize CNTs for the detection of NO_2_ [81]. Sensors based on the PANI/MWCNT composite showed very good sensitivity and fast response time of 50 s when were exposed at different concentrations of NO_2_ gas and a detection limit of 0.05 ppm. In addition, the PANI/CNT composites changed their electronic properties from a p-type to an n-type semiconductor when the samples underwent heat treatment at 80 °C for 24 h; sensors improved the response time to 5.2 s with a detection limit of 0.0167 ppm [82]. This improvement is due to the high permeability of the PANI/CNT compound, which causes a rapid diffusion of the gas through the polymer passageways, the high mobility of the charge carriers of the composite, and the interaction between amino groups and NO_2_ molecules, as shown in Figure 4.

Another research group functionalized CNTs using a carboxylic group to improve the detection of NO_2_ gas. The sensing properties were obtained at different temperatures and NO_2_ concentrations, which obtained the highest sensitivity of 26.88% at a concentration of 100 ppm tested at RT [83]. Jeon et al. fabricated a NO gas sensor based on CNTs functionalized with an amine group, where the response was 50% at RT [84]. Sensing properties based on sulfuric acid-functionalized CNTs have been reported by Ionete et al. The sensors exhibited good response at RT with high sensitivity when exposed to NO and NO_2_ gas at a concentration of 0.04–0.8 ppm [85]. For NO sensing, the sensor showed a response and a recovery time of 255 and 50 s, respectively; whereas, for NO_2_, it exhibited a response and a recovery time of 540 and 420 s, respectively. Finally, carbon nanotubes functionalized with manganese porphyrin have been used for the fabrication of NO_2_ sensors. The sensors were operated at different temperatures with a high sensitivity at temperature of 100 °C [86].

### 3.2. Decorated CNTs

**Figure 5:**
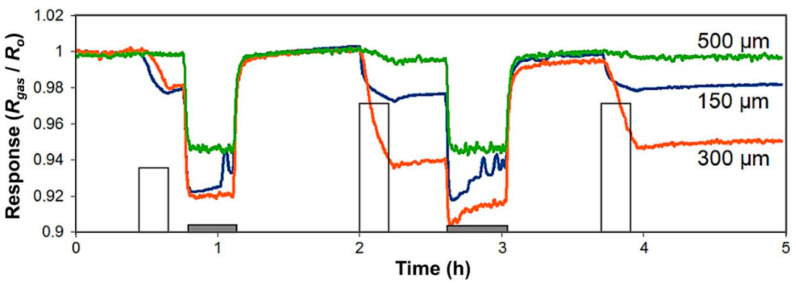
Room temperature detection of NO_2_ for sensors with different CNT lengths. White pulses indicate the exposure to 0.5 ppm, 1 ppm, and 1 ppm of NO_2_ (duration: 15 min). Gray bars indicate the periods of heating at 150 °C that help clean the surface of CNT after being exposed to NO_2_. Heat was not applied after the last exposure cycle, and the baseline was not regained [90].

Another strategy to improve gas sensing is using metallic nanoparticles deposited or supported on the structure of CNTs [87,88]. This strategy has attracted much interest in sensing applications because the catalytic properties of metallic nanoparticles can modify the electronic properties of CNTs using transition metals supported on their surface. The metallic nanoparticles act as catalysts, promoting more reactive sites on their surface for the adsorption of gas molecules. Furthermore, the metal nanoparticles have demonstrated that can bonds strongly with small gas molecules due to their electronic structure and empty orbitals [89]. For example, gold nanoparticles supported on vertically aligned CNTs (VA-CNTs) have been used as gas sensors to detect NO_2_ molecules; CNTs were synthesized using the CVD technique. The tests were carried out at RT using CNTs with lengths of 150, 300, and 500 µm, as shown in Figure 5, to find the best response for 300 µm when the sensors were exposed to NO_2_ gas at different concentrations [90]. This high response to NO_2_ detection was related with the transport unidirectional of the electrical charges and the high effective surface-area-to-volume ratio for CNTs of 300 µm compared with CNTs of 500 µm and 150 µm. However, longer lengths (e.g., 500 µm) of the VA-CNTs could produce a lofty packaging, and this can make the gas detection difficult. In addition, they evaluated the gas sensors at different humidity and found that gas sensors measured at 50% humidity increased the detection of NO_2_ gas. It has reported that relative humidity plays an important role in the electrical conductivity and sensitivity of CNTs [91,92].

**Figure 6:**
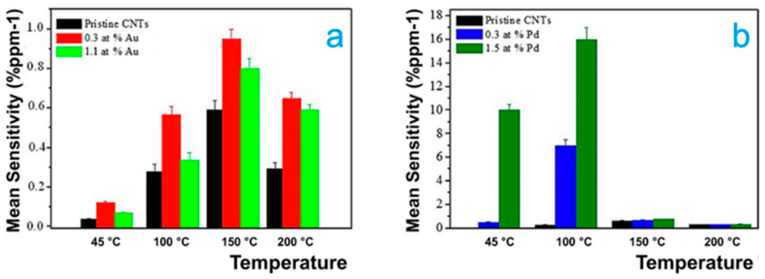
Mean sensitivity of pristine and (**a**) Au- and (**b**) Pd-modified MWCNTs-based sensors toward NO_2_ gas at different sensor operating temperatures in the range 45–200 °C [38].

Ada Fort et al. developed sensors based on CNTs decorated with gold nanoparticles and TiO_2_, which were operated at temperatures below 250 °C. CNTs decorated with nanoparticles showed more activity at low temperatures than pristine CNTs, thus enabling high sensitivity for CNTs decorated with gold nanoparticles with a value of 10% at 12 ppm NO_2_ operating at 240 °C [93]. Dilonardo et al. developed sensors based on CNTs with metallic nanoparticles of Au and Pd deposited on its surface. Sensing tests were carried out at different concentrations of NO_2_ operating at different temperatures (45–200 °C) [38]. Metallic nanoparticles were deposited into CNTs using the electrophoresis technique; these sensors obtained high gas sensitivity, fast response, and low limit of detection, as shown in Figure 6. Using the same strategy, the Pt nanoparticles were supported into CNTs; these sensors were manufactured using the sputtering technique and annealed at a temperature of 500 °C for 1 h under argon atmosphere. Measurements were performed at different concentrations and at various temperatures (25–150 °C), obtaining the best sensing response at a concentration of 2 ppm operating at 100 °C (at least five times higher than pristine CNTs) [94]; in that investigation, it was shown that the performance of sensors based on CNTs were degraded when the sensors were preserved in humid environments. In addition, Mahmood and Naje reported sensors based on Pt nanoparticles deposited into CNTs to detect NO_2_ molecules, which exhibited high sensitivity tested at different temperatures. Their study showed an increase of 4.1 times compared to that of pristine CNTs with a value of 150% at RT [95]. Furthermore, TiO_2_ and Au nanoparticles on CNTs were used for NO_2_ gas sensing. The sensitivity of the sensor was enhanced using pulsed temperature mode, which consisted of variable working temperature using a pulse train [96]. CNTs were decorated with WO_3_ nanoparticles to form WO_3_/MWCNT composite by metal organic decomposition method for NO_2_ gas sensing [97]. The sensors were exposed to NO_2_ gas at different concentrations and measured at RT; the highest sensitivity obtained was in the range of 0.1–0.2 ppm.

Table 1 summarizes the different nanoparticles supported on CNTs that have been used for the sensing of NO_2_ gases. An analysis of sensing properties presented in this table indicates that the lower detection limit is 0.003 ppm detected by the Pt-SWCNTs system operating at 200 °C [87], while at RT, the detection limit is 0.088 ppm using ZnO-SWCNTs [98]. Table 1 also indicates that the Ag-SWCNTs sensor has the lowest time response of 8 s compared with other systems [95]. All these results demonstrate that decorated CNTs with metal nanoparticles can enhance the response sensor due the metal nanoparticles. Thus, the role of metal nanoparticles on CNTs is to accelerate the surface reaction, increase active sites for the adsorption of gas molecules, and improve the electrical properties, which in turn increase the sensitivity to small gas molecules.

### 3.3. Doped CNTs

**Figure 7:**
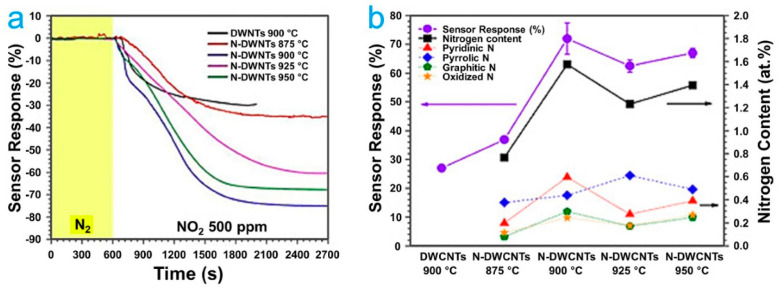
(**a**) sensor responses (%) of all sensors toward 500 ppm of NO_2_ as a function of time. (**b**) sensor response saturation limit (left axis) and the ratio of different types of nitrogen as a function of synthesized temperature [39].

The doping of materials is one of the most used strategies to modify the electrical and electronic properties of CNTs by replacing carbon atoms with heteroatoms. Several elements have been used for the doping of CNTs, which have improved the electrical properties of the CNTs. For example, the detection of NO_2_ on pristine double-walled CNTs and doped with N was studied by Muangrat et al. CNTs were synthesized at different temperatures and doped at different concentrations, where the pristine CNTs diameter was slightly larger than nitrogen-doped CNTs. In addition, they showed that a higher concentration of nitrogen (1.6 at %) decreases the crystallinity of the material. For sensor fabrication, the CNTs powders were dispersed in ethanol using ultrasonication and then deposited by drop casting on a hot ceramic substrate with a temperature of 100 °C. Their results showed that the N-doped nanotube synthesized at 900 °C with 1.6 at % of nitrogen exhibited the best response to NO_2_ gas with a value of 60% more than the pristine CNTs tested at RT [39] (Figure 7). Thus, the previous study demonstrated that the CNTs doped with heteroatoms of N increased the NO_2_ detection, which is related to the high transfer of charge between the CNTs defects and gas molecules.

## 4. Theoretical Studies

In recent years, the theoretical design of nanomaterials has gained great importance. Among the different levels of theory that have been used to study novel materials, approaches based on quantum mechanics can be discarded, e.g., density functional theory (DFT), which has been widely used as a predictive tool for novel materials because its agreement with the experiment is very good [107,108]. It has been widely used to study novel materials in different fields, such as catalysis, electronics, and sensors [109,110,111,112]. In this section, we analyze the theoretical studies developed on CNTs modified through surface functionalization and defect engineering to improve the sensitivity and selectivity to NO_x_.

### 4.1. Decorated CNTs

As previously mentioned, a strategy used to modify the reactivity of CNTs is through surface modification by depositing different atoms or nanoparticles on their surface. In this sense, there are several DFT-based theoretical studies on the reactivity of transition metals-decorated CNTs to NO_x_ gases. For example, the NO adsorption on Pd- and Pt-decorated CNTs was investigated using the PW91 functional [113]. The NO adsorption energy on Pd-decorated CNTs and Pt-decorated CNTs was −1.81 and −2.29 eV, respectively. Very recently, the NO_2_ interaction on Rh_3_M alloys (M = Rh, Ag, Ir, Pd, Pt, and Au) deposited on CNTs was computed employing the BLYP functional [114]. The NO_2_ adsorption energy on RhM-decorated CNTs was between −1.67 and −2.28 eV. These results suggest that the deposition of atoms or nanoparticles on the surfaces of CNTs is a good strategy to increase their surface reactivity. In addition, it can be deduced that transition metals-decorated CNTs can be better candidates for the detection of NO_x_ molecules.

### 4.2. Doped CNTs

Doping has been widely used to modify the structural, electronic, and reactive properties of pristine carbon nanomaterials to NO_x_ gases [115,116,117]. In this same direction, there are numerous studies on the use of doping as a strategy to improve the properties of CNTs with respect to NO_x_ gases. At the theoretical level, various strategies have been used to dope the CNTs. One of the most used routes to dope the CNTs is by substituting a C atom for a heteroatom. For NO molecules, the NO interaction on Al-doped SWNT was recently investigated using the Perdew–Burke–Ernzerhof (PBE) generalized gradient approximation (GGA) [118]. The NO adsorption energy on Al-doped SWCNTs (−1.57 eV) was high compared to pristine SWCNT (−0.09 eV). In another study, the NO molecule interaction on Ni-, Pd-, and Pt-doped SWCNTs, employing the WB97XD functional, was analyzed [119]. The calculated adsorption energy values were −2.47, −3.58, and −3.56 eV for Ni-, Pd-, and Pt-doped SWCNTs, respectively. For the NO_2_ molecule, Table 2 lists the different doping elements used to dope the CNTs by substituting the doping atom for a C atom. All the interaction energies of NO_2_ on doped CNTs were higher than those on pristine CNTs, because for the pristine CNTs, NO_2_ adsorption energies of less than 0.25 eV have been reported. These results clearly show that the doped CNTs are better candidates for NO_2_ detection than pristine CNTs [120,121,122]. This increase in the NO_2_ adsorption energies can be associated to the modification of the electronic and structural properties of doped CNTs with respect to pristine CNTs. In addition, it is clearly demonstrated that when doping occurs in CNTs, the concentration of the doping element substantially determines the properties of the doped CNTs. In this direction, NO and NO_2_ adsorption on CNTs doped with different numbers of Al atoms was investigated using the PBE approximation [118]. It was shown that the NO and NO_2_ adsorption energies tend to increase as the content of Al in the doped CNTs increased, which can be associated to the enlarged active sites on CNTs provided by Al atoms.

More complex doping has recently been investigated such as N_4_ porphyrin-like CNTs with transition metals. The presence of this structure in CNTs provides an increase in their reactivity compared with pristine CNTs. In this direction, the NO adsorption on CoN_4_-CNTs was investigated using the PBE functional. The NO adsorption energy on CoN_4_-CNTs (−1.21 eV) was high compared to those reported for pristine CNT [124]. In another more recent study, the NO and NO_2_ interaction with MnN_4_-CNT was computed employing the PBE approximation [125]. The NO and NO_2_ adsorption energies on MnN_4_-CNT were −2.41 and −1.74 eV, respectively. As in the previous case, the interaction energies of NO and NO_2_ on MnN_4_-CNT were higher than those on pristine CNTs, which shows that N_4_-CNTs with transition metals are better candidates for the detection of these toxic gases than pristine CNTs.

### 4.3. Vacancies

Another strategy used to modify the reactivity of pristine CNTs is through vacancies (Figure 1). As previously documented, vacancy defects substantially modify the electronic, mechanical, and chemical properties of CNTs [126,127]. In this direction, there are some theoretical studies on the reactivity of CNTs with vacancies. For example, Vasylenko et al. investigated the NO interaction on metallic SWCNTs (8,0) with vacancy using generalized gradient approximation [128]. The adsorption energy of NO on metallic SWCNTs (8,0) with vacancy was −2.49 eV, which was higher than reported on pristine CNTs. These results show that SWCNTs with vacancy are better candidates for the detection of NO_x_ gases than pristine CNTs.

## 5. Combined Theoretical and Experimental Studies

A very interesting route for designing novel materials is combining theory and experiment [129,130]. It has been demonstrated that combining experimental results and DFT calculations is very efficient for designing novel toxic-gas sensors [131,132,133]. Therefore, in this section, we analyze the coupled theoretical and experimental studies on developed on CNTs modified through surface functionalization and defect engineering to improve the sensitivity and selectivity to NO_x_. The sensing properties of CNTs decorated with gold nanoparticles to NO_2_ were investigated through coupled theoretical and experimental methods [100]. First, three type of active layers (O_2_-MWNTs, Au (5 Å)-decorated MWNTs, and Au (10 Å)-decorated MWNTs) were exposed to various concentrations of NO_2_. The Au–O_2_-decorated MWNTs sensors detect NO_2_ down to 0.1 ppm. In addition, Au-decorated MWCNTs improve the detection of NO_2_ compared with that of O_2_-functionalized MWCNTs sensors. Finally, the best response to NO_2_ is achieved for Au (5 Å)-decorated MWCNTs sensors. To explain the sensing properties obtained experimentally, DFT calculations were carried out on SWCNTs (5,5) decorated with an Au_13_ nanoparticles in the presence of NO_2_ gas. The Au_13_ deposited on SWCNTs slightly modified the electronic properties of pristine SWCNTs. In addition, a strong interaction (−3.26 eV) between NO_2_ and Au_13_-decorated SWCNTs was observed, which can be associated with a good sensitivity of Au_13_-decorated SWCNTs to NO_2_ gas. In another study, the adsorption of NO_2_ molecules on B- and N-doped CNTs was studied by Adjizian et al. [134]. First, the CNTs doped with B and N were obtained using the CVD technique. The presence of B and N atoms in the structure of CNTs increased the value of the intensity ratio between the D-band and G-band in the Raman spectrum, which increase the density of structural defects modifying the chemical reactivity. The tests were performed using an airtight chamber at concentrations of 0.05, 0.1, 0.2, 0.5, and 1.0 ppm of NO_2_ operating at RT and 150 °C (Table 3). The sensors showed a good response to NO_2_ for both N- and B-doped CNTs operating at both temperatures compared with pristine CNTs. The sensors showed a response at low concentrations with a detection limit of 0.05 ppm increasing with gas concentration. Furthermore, the experimental results demonstrated that the N-doped CNTs are more stable than B-CNTs, which showed the best sensitivity when exposed to NO_2_ gas. Then, they used graphene as a model for the density functional calculations, which demonstrated that the reactivity of pristine graphene is enhanced by doping with B and N.

## 6. Conclusions and Future Directions

This review analyzes the progress of modified CNTs as NO_x_ sensors in the last decade. The different modifications made to the CNTs from the experimental, theoretical, and combined theoretical–experimental perspectives are reviewed. Based on this review, the following conclusions and future directions are proposed.

At the experimental level, CNTs functionalized with conductive polymers, such as PANI and PEDOT, improved the sensor’s response to NO_2_. In addition, the use of metallic nanoparticles supported on CNTs has achieved great progress in the development of NO_2_ gas sensors, which is related to the catalytic spillover effect of the nanoparticles increasing the electron transfer between metal nanoparticles and CNTs. Therefore, sensors based on polymer-modified or metallic nanoparticle-modified CNTs have shown good responses to NO_2_ compared with pristine CNTs because these materials have more reactive sites for the adsorption of the gas molecule. Unfortunately, there is little research on the use of CNTs doped with heteroatoms for gas sensing, which may be due to the difficulty of controlling doping concentration experimentally. Although there has been significant advancement in NO_2_ sensing with these materials, there are challenges to be overcome, such as improving the sensitivity at low concentrations and RT, selectivity, and industrial manufacturing scalable sensors.

Many theoretical DFT-based studies have been developed on modified CNTs as NO_x_ sensors. At the DFT level, different modifications have been investigated on CNTs, such as decorated, doping, and vacancy, in which doping is the most explored. These modified CNTs have shown higher reactivity than pristine CNTs; therefore, they are a good strategy to modify the sensitivity of the CNTs to NO_x_. However, to date, DFT studies have been mainly focused on the sensitivity of modified CNTs to NO_x_. Therefore, it is necessary to investigate the selectivity of the modified CNTs to NO_x_ gases at the DFT level. In addition, feasible approaches (e.g., applying an electronic field) to facilitate the desorption of NO_x_ gases on the modified CNTs should be theoretically investigated in detail.

Coupled theoretical–experimental investigations are a good strategy for designing more sensitive and selective NO_x_ sensors based on modified CNTs. However, in the last decade, these types of investigations have remained scarce. Therefore, investigations combining theory and experiment should be performed to design novel NO_x_ sensors employing modified CNTs.

## Figures and Tables

**Table 1:** Summary of NO_2_ gas sensors based on CNTs decorated with nanoparticles.

Sensor Type	Operating Temperature °C	Limit of Detection (ppm)	Response Time	Recovery Time	Reference
Pt-SWCNTs	200	0.003	<600 s	-	[87]
Pt-MWCNTs	25	1.7	-	-	[99]
Pt-SWCNTs	25–150	2	>180 s	849–1411 s	[94]
Pd-SWCNTs	200	0.009	<600 s	-	[87]
Pd-MWCNTs	25	1.7	-	-	[99]
Pd-SWCNTs	45–200	0.2	<300 s	>1300 s	[38]
Au-MWCNTs	RT	0.1	>600 s	-	[100]
Au-MWCNTs	45–200	0.2	<300 s	>1300 s	[38]
Au-MWCNTs	100–250	5	>30 s	7–4 min	[93]
SnO_2_-MWCNTs	30–200	0.1	<420 s	>8 min	[40]
SnO_2_-SWCNTs	180–380	0.3	<100 s	-	[101]
TiO_2_-SWCNTs	100–250	5	>60 s	6–3 min	[93]
ZnTe-SWCNTs	RT	0.5	-	-	[102]
Rh-MWCNTs	RT	0.05	20 min	-	[88]
Cdots-SWCNTs	RT	0.1	381 s	294 s	[103]
ZnO-SWCNTs	RT	0.088	<220 s	-	[98]
ZnO-SWCNTs	25–300	1	300 s	5–8 min	[104]
Ag-SWCNTs	RT	-	8 s	15 s	[95]
WO_3_-SWCNTs	250–300	0.05	25 min	-	[105]
WO_3_-SWCNTs	RT	0.1	10 min	27 min	[106]

**Table 2:** NO_2_ adsorption on doped CNTs.

Doping Atom	E_ads_ (in eV)	Methodology	Reference
Al	−2.20	B3LYP	[120]
Al	−4.24	BPE	[118]
P	−1.60	B3LYP	[120]
Cr	−2.34	B3LYP	[121]
Mn	−1.82	B3LYP	[121]
Co	−2.36	B3LYP	[122]
Zn	−2.02	B3LYP	[123]
Mo	−3.17	B3LYP	[121]
Tc	−2.06	B3LYP	[121]
Rh	−2.08	B3LYP	[122]
Pd	−2.09	B3LYP	[123]
W	−3.90	B3LYP	[121]
Re	−2.83	B3LYP	[121]
Os	−2.50	B3LYP	[123]
Ir	−2.62	B3LYP	[122]

**Table 3:** Experimental gas sensing responsiveness, S, for nitrogen- and boron-doped nanotubes at an ambient temperature and 150 °C for different gas concentrations. Republished with permission of Elsevier from [134].

Sensor Type	Operating Temperature	NO_2_			
		0.05 ppm	0.2 ppm	0.5 ppm	1.0 ppm
N-CNT	Ambient	−0.75	−2.01	−3.27	−5.5
	150 °C	−0.54	−1.21	−1.87	−2.76
B-CNT	Ambient	0.00	−0.91	−1.39	−1.63
	150 °C	−1.33	−1.98	−3.56	−3.98

## Data Availability

Not applicable.
